# Climate change, 24-hour movement behaviors, and health: a mini umbrella review

**DOI:** 10.1186/s41256-021-00198-z

**Published:** 2021-04-29

**Authors:** Evaline Zisis, Shawn Hakimi, Eun-Young Lee

**Affiliations:** 1 School of Kinesiology and Health Studies, Queen’s University, KHS 307, 28 Division St, Kingston, ON K7L3N6 Canada; 2 Department of Gender Studies, Queen’s University, Kingston, ON Canada

**Keywords:** Population health, Chronic disease, Global warming, Natural disaster, Physical activity

## Abstract

**Background:**

The worsening climate change and alarming prevalence of communicable and non-communicable diseases continue to threat human life and existence. Accumulating evidence suggests that favorable patterns of 24-h movement behaviors, high physical activity, low sedentary behavior, and adequate sleep, may positively contribute to achieving dual benefits of climate change mitigation and disease prevention. The purposes of this mini umbrella review were to summarize the most up-to-date, high-level evidence exploring the relationships between climate change, 24-h movement behaviors, and health and elaborate on the mechanisms linking the three variables of interest.

**Methods:**

A systematic search of electronic databases was performed in PubMed and Google Scholar during March–October 2020. Inclusion criteria were: (1) systematic review; (2) reviewed relationships between climate change and movement behaviors and/or health in any directions; (3) written in English; (4) published in 2010–2020. Narrative synthesis was conducted to highlight the main relationships observed and address the current state of knowledge and priorities for future research. In order to illustrate the potential mechanisms between climate change, movement behaviors, and health, the main results from included systematic reviews were summarized and a conceptual framework was developed for future research.

**Results:**

Based on the evidence from eight systematic reviews published in the past decade, multi-directional (i.e., uni-, bi-, or U-shaped) links were observed between climate change and varying human health outcomes. However, little is understood about the association between climate change and 24-h movement behaviors. Two reviews suggested the negative impact of climate change on sleep and bi-directional relationships between climate change and physical activity/sport. One review included two studies suggesting the unfavorable impact of climate change on sedentary behavior; however, the evidence was limited. Finally, no reviews examined the mechanisms by which climate change, movement behaviors, and health impact one another. Based on the findings of this mini umbrella review, a conceptual framework is proposed that could guide future work to unpack mechanisms between climate change, movement behaviors, and health.

**Conclusions:**

This mini umbrella review highlights the importance of better understanding the mechanisms between climate change, movement behaviors, and health in developing effective mitigation and adaptation strategies to climate change, while paying close attention to vulnerable countries/communities/population groups.

## Background

Since the Industrial Revolution, economic activities have relied on the burning of fossil fuels, which has led to an increase in atmospheric carbon dioxide concentrations and other greenhouse gas (GHG) emissions [1]. As a result, an increased greenhouse effect has led to warming of the earth’s atmosphere. Climate change poses a great threat to several aspects of human life including sustainability [2], economy [3], and health [4]. For example, yearly deaths attributable to climate change are expected to surpass 250,000 between the years 2030 and 2050 [5]. Climate change may also impact human health indirectly by affecting the availability of resources necessary for human life such as local food, safe outdoor environments, or health-related behaviors such as active commuting [6, 7]. Given this startling evidence, minimizing negative impacts of climate change on human health has been a focus of public health [8].

In addition to the threat from climate change to human health, lack of physical activity (PA), sedentariness, and inadequate sleep have been associated with varying health outcomes [9–11]. PA, sedentary behavior (SB), and sleep are three main movement behaviors that occur in a 24-h period, known as 24-h movement behaviors [12]. In general, global populations, particularly those living in rapidly developing and developed countries, have shown low levels of PA (also known as physical inactivity), increased reliance on motorized vehicles, and electronic screen-based home entertainment, and fewer physically demanding professions [13]. These behavioral changes as a result of industrialization and urbanization, along with improved technology and resources have contributed in reducing the physical demands of day-to-day life [14, 15]. Together, all these developments and subsequent lifestyle changes likely led to increased energy consumption and GHG emissions, and have ultimately exacerbated climate change [16].

Though limited to observational studies, the recent time-use epidemiology literature emphasizes that re-allocation of time between PA, SB, and sleep (e.g., re-allocating 30 min of screen time with PA) is favorably associated with weight change [17], body mass index [18], waist circumference [19], obesity [20], executive functioning [21], cardiometabolic health [22], symptoms of fatigue [23], and all-cause mortality [24] in varying age and population groups. In addition to the known health benefits of re-allocating movement behaviors (e.g., replacing 30-min of SB with PA), the Intergovernmental Panel on Climate Change report highlighted that lifestyle behaviors and cultural change have great potential for climate change mitigation and adaptation efforts [25]. Given that climate change mitigation and health promotion efforts can go hand in hand, a better understanding of the associations between climate change, 24-h movement behaviors, and health is important.

Research on climate change and human health has been gaining traction over the past few decades; however, mechanisms between climate change, 24-h movement behaviors, and health inclusively, and the role that 24-h movement behaviors may play in moderating or mediating the relationship between climate change and health are largely unexplored. Better understanding of such complex mechanisms may inform future climate change mitigation and adaptation strategies. The purposes of this mini umbrella review were 1) to summarize the findings of systematic reviews exploring the topics of climate change and 24-h movement behaviors and/or health and 2) to elaborate on the mechanisms linking the three variables of interest (i.e., climate change, 24-h movement behaviors, and health).

## Methods

This review is in a form of mini-review which summarizes “the most salient concepts related to a topic while reporting the most relevant and current findings” [26]. Furthermore, umbrella review was utilized to offer possible solutions and future directions that could address a broad scope of issues related to climate change and movement behaviors and/or health [27]. To conduct a mini umbrella review, peer-reviewed systematic reviews explaining the associations between climate change and 24-h movement behaviors and/or health outcomes published in the past 10 years were systematically examined and summarized, using Preferred Reporting Items for Systematic Reviews and Meta-Analyses (PRISMA) guidelines [28]. Only systematic reviews were considered given the vast scope of the topic and heterogeneity that exist in each variable of interest across different studies with the following definitions for each variable of interest. Climate change is operationalized as one of the biggest issues of our time due to anthropocentric activities, including shifting weather patterns, rising sea levels, greenhouse gases effect, poor air quality and air pollution, patterns and intensities of natural disasters (e.g., extreme rain falls, droughts, floods, storm, bushfires), extreme weather events, and allergens and disease vectors (e.g., ticks, bugs, blackflies, mosquitoes) [29, 30]. Based on the time-use epidemiology thinking in recent years [12, 31], 24-h movement behaviors indicate three key movement behaviors that individuals engage within a 24-h period, which includes PA, SB, and sleep. Additionally, for this review, physical, mental, and social well-being, as well as communicable diseases (CD) and non-communicable diseases (NCD), were considered as health outcomes inclusively [32].

### Literature search

A search for systematic reviews on the topics encompassing climate change, 24-h movement behaviors and/or health was performed (EZ) in PubMed in March 2020 using the following keywords: (“climate change” OR “global warming)” OR (“sleep” OR “PA” OR “physical inactivity” OR “sedentary behav*”) OR “health”. The year range of 2010–2020 was set to identify only the most up-to-date systematic reviews. Google Scholar was searched using similar keywords (SH and EL) to ensure that all relevant reviews were captured. The list was cross validated with the results from PubMed. A top-up search was conducted on June 1, 2020, and expert suggestion was received on October 1, 2020.

### Inclusion criteria

Review articles were deemed eligible for inclusion if the following criteria were met: 1) systematic review; 2) reviewed relationships between climate change and 24-h movement behaviors and/or health (i.e., reviews were deemed eligible if climate change and any of the three 2-h movement behaviors or health were included in their investigation); 3) in English; 4) published in 2010 and 2020.

### Screening process and data extraction

The screening criteria were established a priori (EL). Duplicates were removed and searched reviews were screened based on their titles and abstracts then full-text by two reviewers (EZ and SH). Data screening was supervised by the principal investigator (EL). A data extraction form was developed by the principal investigator (EL) and the main data extractor (EZ) which included study characteristics (e.g., authors, year of publication), number of original articles included in each review, relationships observed, topic of interest, and summary of findings. Data extraction was conducted by the first author (EZ) then verified by the principal investigator (EL).

### Evidence synthesis

Narrative synthesis was conducted (EZ) and verified (SH and EL) to highlight the main relationships observed and to address the current state of knowledge and priorities for future research. In order to illustrate the potential mechanisms between climate change and 24-h movement behaviors and/or health, the main results from the included systematic reviews were summarized and a conceptual framework was developed (EL).

## Results

**Fig. 1:**
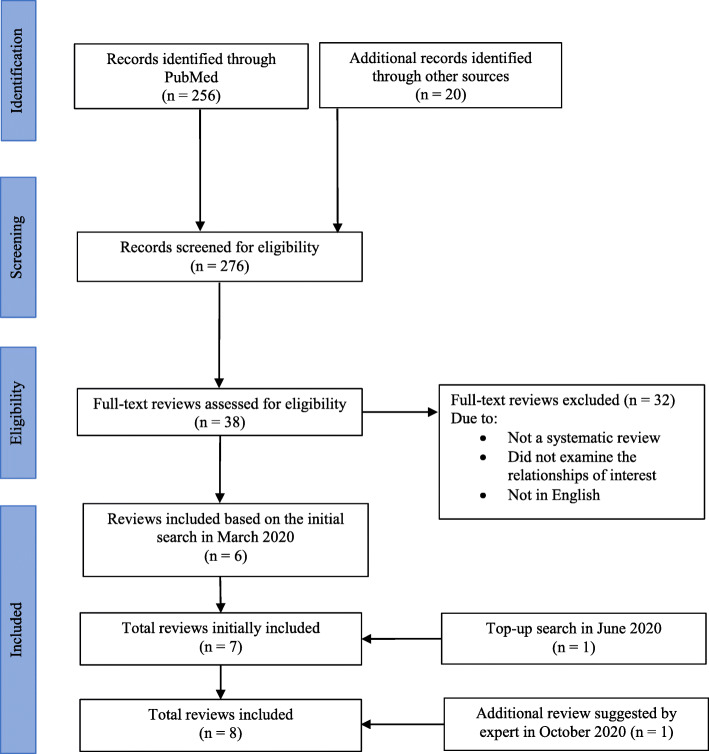
PRISMA flow diagram of the literature reviewing process

Searches in PubMed and Google Scholar yielded 276 articles (Fig. 1). A total of six reviews met the inclusion criteria. Another review was identified through the top-up-search in June 2020 and one additional review was suggested by an expert in October 2020, making up a total of eight systematic reviews included in this review. Of the eight reviews, six evaluated the relationships between climate change and health [33–38], one examined the relationship between climate change and sleep [39], and one evaluated the relationship between climate change and PA [40]. One systematic review [40] was published in the year of 2021; however, it was captured in our search in 2020, thus, included in our review. A descriptive summary and the main findings are presented in Table 1. Systematic reviews included in this review [33–40] had a total of 457, non-mutually exclusive, independent articles and the search time frame ranged from no set time limit up to the year of 2020. None of the included reviews had a specific age group or geographical location of interest; However, one review had a specific focus on developing countries only, given their vulnerability to climate change [35]. All systematic reviews captured from our searches that meet the eligibility criteria were included in evidence synthesis regardless of its quality given the explanatory nature of the present review. In examining the associations between climate change and 24-h movement behaviors and/or health, indicators for each variable of interest used were largely heterogenous across the reviews.

**Table 1:** Summary of systematic reviews included in review

Last name of the first author (publication year)	Search databases	Search period	Search keywords	# of articles in review	Relationship(s) examined	Summary of findings
An et al. 2018 [34]	PubMed, Web of Science, EBSCO, Scopus	– July 2019	(“obesity”, “obese”, “adiposity”, “overweight”, “body mass index”, “BMI”, “weight”, “waist circumference”, “waist to hip”, “waist-to-hip”, “body fat”) AND (“climate change”, “global warming”)	50	Climate change (global warming) and health (NCD focused)	• Four types of relationships identified: 1) global warming and the obesity epidemic are correlated due to common drivers; 2) global warming influences the obesity epidemic; 3) the obesity epidemic influences global warming; 4) global warming and the obesity epidemic influence each other • A fossil fuel-based economy, population growth, and industrialization have contributed to climate change and obesity through land use and urbanization, motorized transportation, and agricultural productivity • Climate change influences the obesity epidemic through food supply and food price and adaptive thermogenesis • The obesity epidemic influences global warming through increased energy consumption (motorized transportation and over nutrition) and greenhouse gas emissions
Bernard et al. 2020 [40]	PsycArticles, CINAHL, SportDiscus, GreenFILE, GeoRef, Scopus, JSTOR, Transportation Research Information Services	–October 2020	(“exercise”, “sport”. “walk”, “biking”, “physical activity”) AND (“climate change”, “natural disaster”, “greenhouse”, “flood”, “extreme weather”, “drought”)	63	Climate change and movement behavior (physical activity)	• There is a bidirectional relationship between climate change and physical activity and sport practices • Air pollution is associated with a less time spent participating in physical activity and more time spent in sedentary time • Extreme weather conditions including heavy precipitation and heat waves lead to decreased active transportation and leisure physical activity • Natural disaster experience is associated with lower levels of physical activity and less active travel • Active transportation reduces carbon dioxide and other greenhouse gas emissions • Participation in sport is related to a larger carbon footprint, particularly in elite sports. This carbon footprint may be reduced through interventions such as carpooling for transportation • Leisure physical activity and sport infrastructure and facilities may help communities’ recovery following a natural disaster • In the future, sports organizations and facilities will have to prepare, and make accommodations for climate change
Cheng and Berry [33]	Medline, Web of Science, GEOBASE, grey literature (e.g., IPCC, WHO)	January 2000–March 2012	(“climate”, “climate change”, “adaptation”) AND (“health status”, “public policy”, “health co-benefits”, “health risk”, “public health”)	22	Climate change and health (NCD and mental health focused)	• Climate change adaptation strategies involving increased social capital have been shown to have health benefits and reduce vulnerability to negative health impacts of climate change • Increased social capital also has potential health risks (by increasing fear and misconceptions within social networks) for facing climate change and must be further studied to improve understanding of risks and benefits • Urban design and planning strategies, such as green spaces, walkable neighborhoods, and bike paths, can have a positive effect on both public health and climate change mitigation • Poor urban planning (e.g., urban heat island effect) can increase health risks in the era of climate change
Franchini and Mannucci [37]	PubMed	–November 2014	“climate change”, “climate variability”, “global warming”, “meteorological factors”, “weather”, “atmosphere”, “heat waves”, “extreme weather”, “ambient air pollution”, “outdoor”, “particulate matter”, “PM”, “air pollutants”, “mortality”, “human health”, “health effects”, “infectious disease”, “diarrheal disease”, “cardiovascular disease”, “ischemic heart disease”, “cancer”, and “respiratory disease”.	77	Climate change and health (both NCD and CD focused)	• Extreme weather events caused by global warming, including heat waves, wildfires, and drought, lead to increased rates of morbidity and mortality in affected regions. For example, there is a link between higher incidence of myocardial infarction and higher temperature • Decreased winter temperatures caused by global warming in mid-lateral regions could lead to health benefits such a decrease in mortality from respiratory and cardiovascular diseases, however, the negative health consequences of rising temperatures have been shown to far outweigh these potential positive ones • Desertification and droughts related to climate change limit low-income countries’ abilities to maintain adequate food production and clean drinking water, leading to poorer health outcomes and increased death rates • Diarrheal diseases will likely increase due to climate warming; droughts, massive rainfall and increasing temperatures as these reduce the availability of safe drinking water • Increased temperatures also increased risk for cardiovascular and respiratory dysfunctions, due to increased concentrations of air pollutants and ozone • Higher levels of carbon dioxide and higher temperatures resulting from global warming are expected to increase the prevalence of allergic diseases globally • Through changes in temperature, rainfall patterns, and extreme climate events, global warming is expected to increase the spread of infectious and vector-borne diseases • There is a relationship between climate change, mean temperature increase, and humidity variations, and visits to the emergency room due to atrial fibrillation, renal colic, and psychiatric issues
Levi et al. [36]	PubMed, EMBASE, SCOPUS	January 2000–June 2017	Focused on i) “heat-related illness”, “cardiovascular”, “respiratory and kidney diseases”, ii) “traumatic injuries”, “acute death”; iii) “vector-borne diseases” or “vectors distribution”. “climate change”, “worker*” AND AND (“health” (health OR injur* OR disease*))	165	Climate change (temperatures) and health (both NCD and CD focused)	• There is an inversed U-shaped relationship between maximum daily temperature and daily injury claims • Increased temperatures put outdoor workers at greater risk for vector-borne infectious disease • When daily temperature exceeds 32 degrees Celsius, daily labor productivity is reduced by up to 14% in agriculture and construction sectors • Overall, as temperatures increase due to climate change, worker health and productivity in outdoor professions is expected to decline
Rataj et al. [35]	Medline, Embase, Web of Science, PsycINFO, CAB Direct, PILOTS; Hand search in Global Environmental Change and Climatic Change journals; Google Scholar, WHO’s Virtual Health Library	–April 2014	Direct link	17	Climate change (extreme weather events) and health (NCD and mental health focused)	• Following a natural disaster experience, there are increased rates of post-traumatic stress disorder, injury, depressive disorder, and anxiety disorder • Risk factors for these health problems following natural disasters consistently included prior traumatic events, female sex, higher age, poor health status, and witnessing death or dead bodies
Rifkin et al. [39]	PubMed, Scopus, Cochrane databases	1980–2017	Direct link	16	Climate change and movement behavior (sleep)	• Increased temperatures lead to decreased time and quality of sleep • A 1-degree Celsius deviation in monthly night-time temperatures was associated with an increase of three nights of insufficient sleep per 100 people • Studies examining the relationship between wildfires, floods, and sleep found that people exposed to these natural disasters had disrupted sleep following the events • Decreased sleep time and increased sleep disruption are common and appear to effect more vulnerable populations (i.e., elderly and low-income persons) more severely
Veenema et al. [38]	PubMed, CINAHL, Embase, Scopus, Web of Science	2006–2016	(“climate change”, “climatic processes”, “El Niño”, “global warming”, “Disasters”, “natural disasters”, “floods”, “flash floods”, “coastal floods”, “flooding”, “cyclone”, “hurricane”, “heavy rainfall/precipitation”, “sea-level rise”) AND (“health” (“population”, “public”, OR “community)))	47	Climate change (water disasters) and health (NCD, CD, and mental health focused)	• Climate change-related water disasters directly impact human health by causing drowning, electrocution, cardiovascular events, and mental health effects • Displacement by flooding may put individuals under close quarters with potentially unsanitary living conditions which perpetuate the spread of infectious diseases • Increases in water temperature, precipitation frequency and severity and other water disaster-caused consequences result in increased waterborne, vector-borne, and zoonotic diseases • Climate change-related water disasters are also associated with negative psychiatric and mental health outcomes such as post-traumatic stress disorder • Climate change-related water disasters likely disproportionately affect individuals in vulnerable positions (e.g., low income, % of minority residents, lower education, lack of English fluency, low take up of medical services, age, disability status). • Lower income countries are more susceptible to water-related disasters with poorer post-disaster outcomes compared to higher income countries • Disruption in food or water sources post water-related disasters can lead to chronic community malnutrition • Water-related disasters also disrupt health infrastructure and affect the continuity of healthcare services during the recovery period, and this is hampered by a lack of health services and adequate health professionals in affected communities

*CD* Communicable diseases, *NCD* Non-communicable diseases

### Climate change and health

Of six reviews [33–38], three reviews [34, 36, 37] evaluated the effects of climate change on physical health outcomes. A bi-directional relationship between global warming and obesity was noted in one review [34], describing that global warming has a negative impact on the obesity epidemic through food supply/price and adaptive thermogenesis (i.e., reduced seasonal exposure to colder climate decreases energy expenditure and contributes to adiposity). Conversely, global warming is exacerbated due to obesity epidemic through increased energy consumption and GHG emissions [34]. Furthermore, extreme weather events caused by global warming (e.g., heat waves, wildfires, drought), led to increased morbidity and mortality [37]. Rising temperatures also increased risk for cardiovascular and respiratory dysfunctions, due to increased concentrations of air pollutants and ozone [37]. Moreover, changes in temperature and rainfall patterns caused by global warming are expected to increase the spread of infectious diseases [36, 37]. Increased temperatures due to climate change was also shown to impact occupational health whereby there is an inverse U-shaped relationship between maximum daily temperature and daily injury claims among outdoor workers [36].

Two reviews focused on the relationships between climate change-related indicators and mental health. One of these reviews [35] found that experiences with natural disasters due to climate change are associated with increased rates of post-traumatic stress disorder, injury, depressive disorder, and anxiety disorder. In addition, the review [35] suggested that climate change-related natural disasters disproportionately affect women, individuals with older age and/or poor health status, and those who witnessed death/dead bodies. Another review [39] indicated that rising temperature and extreme weather events due to climate change are associated with impaired sleep due to fear or depression.

One review [33] prospectively examined how public health and climate change mitigation strategies produce both health and environmental benefits and risks. Results showed that increased social capital has potential health risks by increasing fear and misconceptions within social networks about climate change. The review also suggested that urban design and planning strategies as a response to climate change (e.g., expanding green spaces, walkable neighborhoods, bike paths) can have a positive influence on both public health and climate change mitigation while poor urban planning (e.g., urban heat island effect) can increase health risks.

One review [38] specifically examined the impact of climate change-related water disasters on population health. The review highlighted that people in a vulnerable situation (e.g., living in poverty or unstable dwellings, lack of access to health care) are at an increased risk for mortality and morbidity. For instance, among residents in Southeast Florida, rising sea levels were particularly dangerous for those who are low-income, lower education levels, non-English speaker, older age, visible minority, or those with disability [41].

### Climate change and 24-h movement behaviors

One review evaluated the relationship between climate change and sleep [39] and another review examined the relationship between climate change and PA [40]. Rifkin and colleagues [39] investigated how changes in temperatures, extreme weather events, and natural disasters impact human sleep. Findings from their review showed that increased temperatures lead to decreased sleep quality. Additionally, people who experienced natural disasters were more likely to experience disrupted sleep following the events. In another review [40], a bi-directional relationship between climate change and PA/sport was identified. Varying indicators of climate change were associated with low PA with disproportionate impact on individuals with chronic diseases and older people. The review also suggested that PA contributes to intensifying or mitigating climate change through active/passive modes of commuting and subsequent reductions/increase in GHGs. It was also discussed that access to PA/sport infrastructure may be important factors for recovery in communities affected by natural disasters; however, at the same time, mega sporting events, the professional sport industry, and related air travels by athletes and spectators are major sources of GHGs.

No systematic reviews were focused on the associations between climate change and SB; however, one review on climate change and PA [40] included two studies which examined the unfavorable impact of climate change related indicators on sedentary time. Specifically, one study observed a high level of sedentary time among those who experienced Hurricane Ike, as a symptom of post-traumatic stress compared to non-disaster samples [42]. Another original study that was included in the review [40] showed higher levels of sedentary time in the areas of Beijing that exhibit highly concentrated air pollution [43].

### Climate change, 24-h movement behaviors, and health

None of the identified reviews examined the relationship between all three variables of interest (i.e., climate change and 24-h movement behaviors and/or health) together. One paper [39] alluded to the emerging threats of climate change on sleep duration and interruption, and consequently, increased risk for NCDs given the strong evidence suggesting the association between shortened sleep duration and NCD-related burden. However, Rifkin and colleagues [39] highlighted that the evidence identifying clear links between climate change, sleep, and health remains an important gap requiring attention in future research. The importance of better elucidating the mechanisms between climate change and movement behaviors and/or health has also been highlighted in another review [40]. Specifically, it was noted that increasing incidence of vector-borne diseases such as Lyme disease or Malaria may negatively influence outdoor or occupational PA or active travel.

**Fig. 2:**
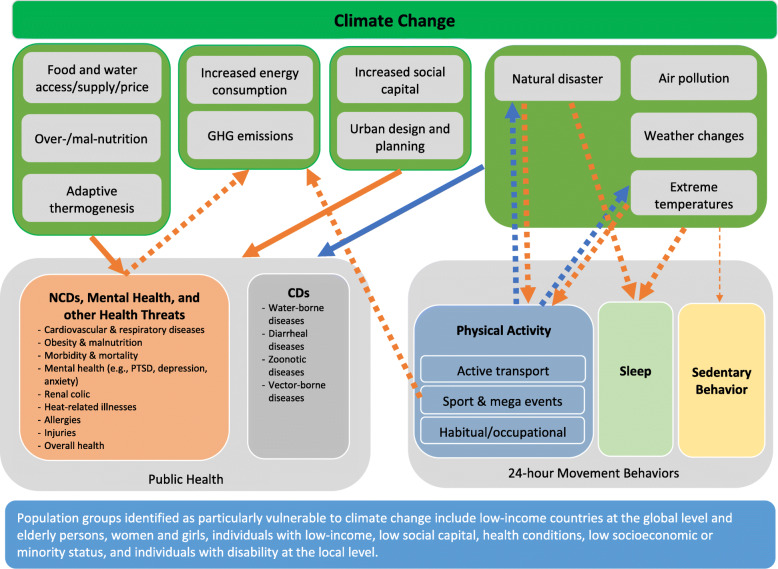
A conceptual framework of directionalities and pathways between climate change, 24-h movement behaviors, and health. *Note*: The top circle indicates climate change and below, indicators of climate change that act as both causes and consequences mentioned in each review are listed. From eight reviews included [35–42], identified, direction and nature of the relationships between indicators of climate change and varying movement behaviors and/or health outcomes are indicated using arrows (direction) and colored lines (blue lines indicate favorable associations and orange lines indicate unfavorable associations). Furthermore, thick solid lines indicate consistent associations (consistent findings in ≥ two reviews) while thick dotted lines (one review) indicate suspected associations. Narrow dotted lines indicate speculated, but largely unknown associations (no reviews but independent studies). Population groups that are identified as particularly vulnerable are described in the blue box at the bottom. CD: Communicable diseases; NCD: Non-communicable diseases; PTSD: Post-traumatic stress disorder

Based on this mini umbrella review, a conceptual framework outlining potential pathways between climate change, 24-h movement behaviors, and health is proposed (Fig. 2) to provide a greater understanding of these multifactorial, multi-directional, and complex relationships. Fig. 2 represents the results from each systematic review article included in this work. Briefly, there are multi-directional relationships between climate change and health, a potential uni-directional relationship between climate change and NCDs via sleep, and bi-directional relationships between climate change and PA. Evidence clarifying the relationship between climate change and SB appears to be largely lacking and warrants future work; however, the potential link is indicated based on previous literature [42–44]. Though the relationships between climate change, 24-h movement behaviors, and health cannot be clarified through this review, individual relationships between climate change and health [35, 36], climate change and 24-h movement behaviors [39, 40], and 24-h movement behaviors and health [9–11] were speculated. A clear mechanism linking the three variables of interest appears to be an important gap requiring further research. In addition, other human behaviors that occur on a day-to-day basis, for example, dietary habits, eco-friendly behavior, or consumer behavior, may also explain the links between climate change and health; therefore, future research may expand this line of inquiry beyond 24-h movement behaviors.

## Discussion

Based on the evidence from eight systematic reviews published in the past decade, a multi-directional link between climate change and human health was observed. Specifically, uni-directional [35, 37, 39], bi-directional [33, 34, 38], and U-shaped associations [36] were observed between climate change and varying human health outcomes. Less is understood about the association between climate change and 24-h movement behaviors. Two reviews suggested the negative impact of climate change on sleep [39] and bi-directional relationships between climate change and PA/sport [40] with partial evidence on SB. No reviews examined the mechanisms by which climate change, 24-h movement behaviors, and health influence one another.

Of the research exploring the impact of climate change on human health, there appears to be a general consensus that climate change is expected to negatively impact various aspects of human health [35, 37–39]. It is also important to note that climate change will disproportionately generate greater risks for low- and middle-income countries (LMIC) [38, 45]. In LMICs, economic loses are largely uninsured (approximately 99%), as such the economic impact of climate change is greater than it is in high-income countries, leading to poorer health outcomes for their citizens. This is because there is less resiliency spending and generally poorer health infrastructure; therefore, when climate change-related disasters happen, it is more difficult for LMICs to recover from and adapt to the changes [45]. It is also reported that at the local level, elderly and low-income individuals [38, 39], individuals with low social capital [33], women and girls [35], and individuals with health conditions [35], low socioeconomic or minority status, as well as individuals with disability [38] are more likely the be more vulnerable to climate change than their respective counterparts. Therefore, climate change mitigation and adaptation strategies should pay close attention to population countries/communities/groups that are more vulnerable to climate change.

Limited, yet apparent associations between climate change and 24-h movement behaviors, specifically sleep [39] and PA/sport, [40] were observed. The relationship reported between climate change and sleep was uni-directional of which increased temperatures and natural disasters due to climate change diminishes and interrupts sleep, especially among low-income and elderly persons [39]. Bi-directional associations between climate change and PA/sport [40] indicated that climate change disproportionately impacts PA in the global population. At the same time, PA can mitigate or exacerbate climate change. Specifically, consistent, negative impacts of air pollution, extreme temperatures, and natural disasters on PA was reported. However, the impact of extreme temperatures on people’s ability to engage in PA, particularly in outdoor activities, can vary by geographical location. For instance, people living in the north temperate zone (e.g., Canada) may not be affected at a same rate as those in the south temperate zone (e.g., Australia) [25, 40]. Such “place effects [46]” and their potential mediating role between climate change and health are being increasingly discussed in the literature [46, 47].

While preliminary evidence suggests that seasonal variation may influence SB [48], no reviews were focusing on exploring SB in relation to climate change. Only two original studies included in the review on climate change and PA [40] reported a negative impact of climate change-related indicators on SB [42, 43]. Given the co-dependent relationship between PA and SB, combined with the well-known health impacts of SB [49], it is important to clarify the potential bi-directional relationships between climate change and SB. In particular, screen time, the most common form of SB that is known to negatively impact health [50] is of great interest. It is projected that the Information Technology sector from powering internet servers and charging personal individual devices will consume as much a 20% of the world’s electricity by 2030 [51]. In the era of climate change, supporting individuals to adjust their time-composition of 24-h movement behaviors so that they engage less in ‘carbon-intensive’ screen-based sedentary behaviors (e.g., streaming movies online), and instead engage in more emission-free SBs (e.g., board games, reading) or varying types of PA should be a priority [52]. Future studies should further explore this relationship in order to provide an understanding of whether this relationship exists, the directions of this relationship, and the possible pathways through which PA and SB interact with each other, and influence and are influenced by climate change.

This review suggests potential directions and pathways between climate change, 24-h movement behaviors, and health and provides a conceptual framework (Fig. 2) that could guide future research on the topic. Some limitations should be noted. This review only included systematic reviews in English and published between 2010 and 2020. In addition, only a limited number of keywords was used in literature searches; therefore, it is possible that reviews specific to one form of disease (e.g., vector-borne disease) or 24-h movement behavior (e.g., active transport, screen time) may have been missed; however, this is unlikely because we also conducted multiple searches on Google Scholar to cross-reference results and additional work was found via expert’s suggestion. Finally, assessment of methodological quality/critical appraisal for each systematic review was not conducted given the broad scope and exploratory nature of the present review.

## Conclusions

This mini umbrella review highlights the importance of better understanding the mechanisms between climate change, 24-h movement behaviors, and health to develop effective mitigation and adaptation strategies to climate change. The impact of climate change on human health could be intensified or buffered by 24-h movement behaviors; however, this requires further investigations to be more conclusive. It is also noteworthy to mention that, in support of previous literature, the findings of this review build on the assertation that promoting healthy movement behaviors can address two major pressing issues of our era, climate change and human health. By considering knowledge pertaining to climate change and health and exploring factors that are linked with both climate change and health, such as 24-h movement behaviors, human health can be better protected through climate change mitigation and adaptation efforts. In making such efforts, it is important to consider countries/communities/population groups that are most vulnerable to climate change.

## Data Availability

Not applicable.

## Abbreviations

CD: Communicable diseases
LMIC: Low- and middle-income countries
NCD: Non-communicable diseases
PA: Physical activity
PRISMA: Preferred Reporting Items for Systematic Reviews and Meta-Analyses
SB: Sedentary behavior
